# Association between Apolipoprotein E gene polymorphism and the tortuosity of extracranial carotid artery

**DOI:** 10.3389/fneur.2025.1498613

**Published:** 2025-05-09

**Authors:** Jun-Wei Wang, Xin Li, Liu-Xi Lu, Jun-Lin Chen, Yong-Tao Yang, Ge Jin

**Affiliations:** Department of Neurology, The Fifth People’s Hospital of Chongqing, Chongqing, China

**Keywords:** ApoE, gene polymorphism, tortuosity, extracranial carotid artery, correlation

## Abstract

**Background:**

It is widely recognized that the Apolipoprotein E (ApoE) exhibits a significant association with dyslipidemia and atherosclerotic cardiovascular disease (ASCVD). The tortuous extracranial carotid artery (ECA) is a frequently encountered vascular morphological anomaly that may be associated to ischemic cerebrovascular disease. The purpose of this study was to investigate the association between ApoE gene polymorphism and the tortuosity of ECA.

**Methods:**

The clinical data and ApoE genetic test of inpatients who underwent head and neck DSA or CTA at our department between June 2020 and January 2024, were retrospectively analyzed. The tortuosity index (TI) of the ECA was measured and calculated. The included patients were analyzed using two grouping methods based on TI of the ECA: three groups determined by the tertile distribution and two groups based on the median distribution. Multivariate logistic regression analysis and Spearman rank correlation analysis were employed to investigate the correlation between ApoE genotypes and ECA tortuosity.

**Results:**

A total of 238 patients were included in the study. The lowest tertile, the middle tertile and the highest tertile of TI distribution encompassed 91 cases (38.2%), 65 cases (27.3%) and 82 cases (34.5%) respectively. On the other hand, there were 127 cases (53.4%) in the low median group and 111 cases (46.6%) in the high median group. Due to the rarity of the three genotypes (ε2/ε2, *n* = 4; ε2/ε4, *n* = 1; ε4/ε4, *n* = 1), they were excluded for further statistical analysis. After adjusting for all covariates, the genotype ε3/ε4 continued to show an independent correlation with ECA tortuosity in the tertile groups (adjusted odds ratio = 0.469, 95% confidence interval: 0.242–0.969, *p* = 0.025). The Spearman’s rank correlation analysis revealed a significant negative correlation between the TI of ECA and ApoE gene polymorphism (in sequential order: ε2/ε3, ε3/ε3, and ε3/ε4) (*r_S_* = − 0.149, *p* = 0.023).

**Conclusion:**

Our study suggested that the ε2 allele may be associated with the increased tortuosity of ECA, whereas the ε4 allele may leads to be a protective factor. The ε3 allele, as the most prevalent wild-type in human, has not exert a significant influence on ECA tortuosity.

## Introduction

Apolipoprotein E (ApoE) is expressed primarily by the liver parenchymal cells in the human body and is a major apolipoprotein found in plasma. It exhibits genetic polymorphism, consisting of three alleles (ε2, ε3, and ε4), which combine to form six different genotypes (ε2/ε2, ε2/ε3, ε2/ε4, ε3/ε3, ε3/ε4, and ε4/ε4) (1, 2). The above three alleles (ε2, ε3, and ε4) encode three ApoE isoforms (E2, E3, and E4), which play different roles in maintaining cholesterol metabolic balance. ApoE4 is associated with various diseases, including hyperlipidemia (3), atherosclerosis (4), Alzheimer’s disease (5, 6) coronary atherosclerotic heart disease, and ischemic stroke (7). Extracranial carotid artery (ECA) tortuosity is considered an age-related degenerative change, but its underlying mechanism is still incompletely clear. Previous study has shown severe ECA tortuosity is associated with hemodynamic changes and transient ischemic attack, which commonly arisen from atherosclerotic stenosis (8). Furthermore, the obvious ECA tortuosity increases the challenge of endovascular treatment (9), and may leads to poor prognosis of anterior circulation ischemic stroke patients who without undergoing interventional procedure (10).

Although ECA tortuosity and ApoE are both associated with cerebrovascular disease, the causal relationship between them is currently unknow yet. The aim of this study is to investigate the relationship between ECA tortuosity and ApoE gene polymorphism, and to providing more clues and evidence for the pathogenesis of ECA tortuosity.

## Materials and methods

### Patients

The clinical data and ApoE gene tests of inpatients, who underwent the head and neck digital subtraction angiography (DSA) or computed tomography angiography (CTA) in our department from June 2020 to January 2024, were retrospectively analyzed. The clinical data comprised demographic information and the cerebrovascular disease’s common risk factors. The tortuosity index (TI) of ECA for each patient were measured and calculated. Referring to the methods in previous literatures (11, 12), the included patients were divided into three groups based on the tertile distribution of ECA TI and two groups based on the median distribution. We compared the differences in clinical data and ApoE genotypes between these groups.

The inclusion criteria for this study were hospitalized patients who had completed both head and neck CTA/DSA and ApoE genotyping, without restriction based on disease entities. The exclusion criteria were defined as follows. (1) patients with poor imaging quality of CTA or DSA which could not complete the measure and calculate for the tortuosity index. (2) patients with the bilateral ECA occlusion. (3) patient is under 18 years old. This study was designed for retrospective research, and the formal consent from patients was not required. All research procedures involving human participants adhered to the ethical standards of the Declaration of Helsinki of 1964 and its subsequent amendments or similar ethical standards.

### Acquisition of ECA imaging

The DSA was performed using an Allura xper FD20 X-ray system (Philips, Netherlands), while the CTA was performed using a Brilliance 64 row 128 slice spiral CT (Philips, Netherlands). The three-dimensional volume rendering of the head and neck arteries were achieved using the Philips Extended Brilliance^™^ workstation (Philips, Netherlands). Both DSA and CTA procedures were all using non-ionic contrast agents (iohexol, iopamidol or iodixanol) for imaging. The informed consent form for DSA or CTA procedures were signed by the enrolled patients or their next of kin.

### Quantification of tortuosity index

**Figure 1:**
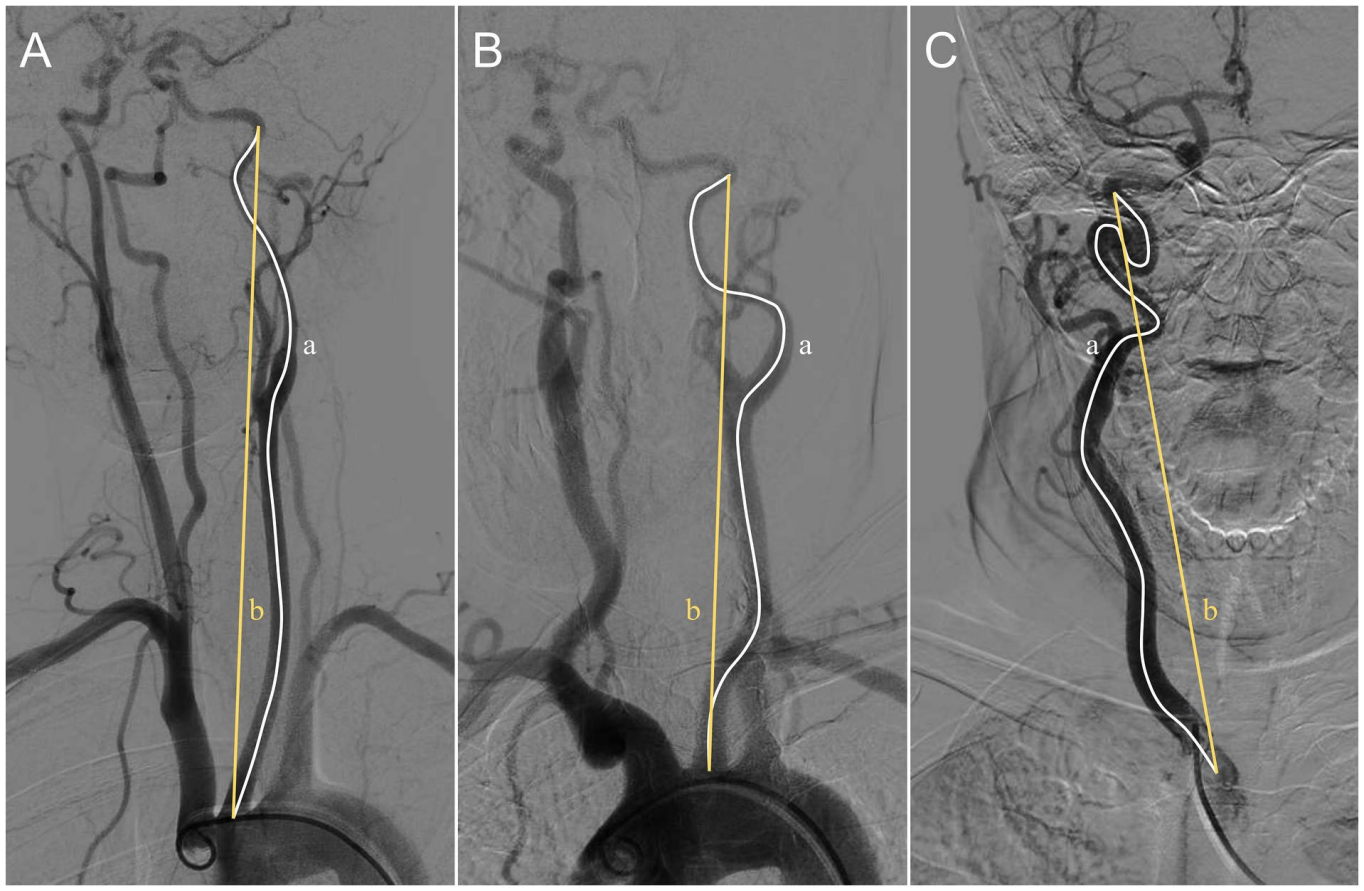
The actual length (white line) and straight length (yellow line) of the left ECA **(A,B)** and the right one **(C)**. Based on the calculations using the formula (TI = [a/b−1] × 100), the tortuosity of ECA in **A** is mild (TI = 4), while that in **B** is moderate (TI = 15), and that in **C** is severe (TI = 36). ECA, extracranial carotid artery; TI, tortuosity index.

The DICOM document of CTA and DSA images were downloaded and collected from picture archiving and communication system (PACS). The tortuosity index of the ECA was measured and calculated by two independent experts in cerebrovascular disease imaging using Sante DICOM Viewer 3D software (Sante soft LTD, Nicosia, Cyprus). After loading DICOM files or selected JPEG format images into the Sante DICOM Viewer software, the anteroposterior view of 3D volume-rendered images for bilateral ECA were choose to measure. The proximal end for measure was the bifurcation point of the aortic arch in the left ECA or the brachiocephalic trunk in the right ECA. The distal end of measurement was the ECA located at the entrance of the carotid canal. A line measurement tool was used to measure the actual length from the proximal end to the distal end along the curvature of the ECA, and a linear distance tool was used to measure the straight length between the two points. The calculation formula for the TI of ECA is as follows. TI = [actual length/straight length−1] × 100. Since relative numbers cannot be averaged, the higher value obtained from the bilateral ECA calculations was taken to represent the TI of the patient. The specific measurement and calculation methods refer to the previously published literature (13, 14) (Figure 1).

### Detection of ApoE genotype

After extracting DNA from venous blood of patients, the ApoE genotypes was detected and analyzed by Fascan-48E Multichannel Fluorescence Quantitative Analyzer and SNP-U4 Human ApoE Gene PCR Detection Kit (TIANLONG Technology Ltd., Xi’an city, CHN). The genotype of each subject was one of six possible results: three homozygotes (ε2/ε2, ε3/ε3, ε4/ε4) or three heterozygotes (ε2/ε3, ε2/ε4, ε3/ε4).

### Statistical analysis

All the data were statistically processed by using SPSS software (version 20.0, IBM Corporation, Armonk, NY). The Kolmogorov Smirnov test and Shapiro Wilk test was used to examine the normal distribution of continuous variables, and the Levene test is used to test the homogeneity of variance. The continuous variables that follow a normal distribution were described as Mean (SD), and inter group comparisons were performed by using the analysis of variance or the student’s t test. The continuous variables with skewed distribution were described as Median (IQR), and intergroup comparisons were performed by using Kruskal Wallis rank-sum test or Mann Whitney U test. The categorical variables were described as *n* (%), and inter group comparisons were conducted by using chi-square test, Fisher’s exact test, or Kruskal Wallis rank-sum test. The multivariate analysis was performed by using the ordinal logistic regression and binary logistic regression. The odds ratio (OR) and 95% confidence interval (CI) were calculated to analyze the independent risk factors for ECA tortuosity. The spearman correlation coefficient (*r*_s_) was used to analyze the correlation between ApoE gene polymorphism and the degree of ECA tortuosity. *p* < 0.05 indicates the statistically significant.

## Results

### Univariate analysis

A total of 238 patients with ischemic cerebrovascular disease or those with its risk factors were included in this study. According to the tertile distribution of ECA TI, there were 91 cases (38.2%) in the TI < 13 group, 65 cases (27.3%) in the TI 13–19 group, and 82 cases (34.5%) in the TI > 19 group. According to the median distribution of ECA TI, there were 127 cases (53.4%) in the TI ≤ 15 group and 111 cases (46.6%) in the TI > 15 group. The age, sex, hypertension, diabetes, total cholesterol (TC), triglyceride (TG), low-density lipoprotein cholesterol (LDL-C), atrial fibrillation, smoking history, drinking history, and previous stroke history were analyzed and compared.

Among the 238 patients included, the distribution of ApoE genotypes is as follows: 4 cases of ε2/ε2 (1.68%), 40 cases of ε2/ε3 (16.8%), 1 case of ε2/ε4 (0.42%), 143 cases of ε3/ε3 (60.1%), 49 cases of ε3/ε4 (20.58%), and 1 case of ε4/ε4 (0.42%). Due to the rarity of genotypes ε2/ε2, ε2/ε4, and ε4/ε4, the six cases were excluded for further statistical analysis as refer to a previous research (15).

There were significant differences (*p* < 0.05) in age, female gender, and smoking history between the tertile groups and the median groups. In addition, there were significant differences in alcohol consumption between the median groups and in atrial fibrillation between the tertile groups. As for the genotypes of ApoE, ε3/ε4 (*p* = 0.029) showed statistically difference in the tertile groups for ECA TI. In contrast, no statistically significant differences were observed in either grouping methods for ε2/ε3 and ε3/ε3 (Table 1).

**Table 1:** The characteristics of clinical data and ApoE genotypes between the tertile and the median groups for ECA TI.

Factors	The tertile groups of TI			*p* value	The median groups of TI		*p* value
	TI < 13	TI 13–19	TI > 19		TI ≤ 15	TI > 15	
	(*n* = 91)	(*n* = 65)	(*n* = 82)		(*n* = 127)	(*n* = 111)	
Age in years, mean (SD)	63.9 (11.1)	69.4 (10.6)	71.7 (10.6)	<0.001	65.2 (11.1)	71.4 (10.5)	<0.001
Female, *n*(%)	18 (19.8)	21 (32.3)	57 (69.5)	<0.001	29 (22.8)	67 (60.4)	<0.001
Hypertension, *n*(%)	70 (76.9)	54 (83.1)	67 (81.7)	0.585	100 (78.7)	91 (82.0)	0.531
Diabetes mellitus, *n*(%)	34 (37.4)	32 (49.2)	27 (32.9)	0.121	53 (41.7)	40 (36.0)	0.369
TG, median (IQR)	1.33 (1.43)	1.3 (1.17)	1.29 (0.8)	0.923	1.30 (1.20)	1.29 (0.85)	0.710
TC, median (IQR)	4.46 (1.65)	4.28 (1.69)	4.42 (1.8)	0.214	4.46 (1.66)	4.36 (1.57)	0.966
LDL-C, median (IQR)	2.78 (0.97)	2.71 (1.34)	2.68 (1.44)	0.348	2.76 (1.00)	2.69 (1.23)	0.934
Atrial fibrillation, *n*(%)	7 (7.7)	0 (0)	5 (6.1)	0.048	7 (5.5)	5 (4.5)	0.775
Smoking, *n*(%)	51 (56.0)	25 (38.5)	13 (15.9)	<0.001	66 (52.0)	23 (20.7)	<0.001
Alcohol drinking, *n*(%)	23 (25.3)	15 (23.1)	7 (8.5)	0.978	32 (25.2)	13 (11.7)	0.008
Stroke history, *n*(%)	11 (12.1)	7 (10.8)	8 (9.8)	0.965	15 (11.8)	11 (9.9)	0.682
ApoE genotype, *n*(%)							
ε2/ε2	0 (0)	3 (4.6)	1 (1.2)	/	1 (0.8)	3 (2.7)	/
ε2/ε3	12 (13.2)	9 (13.8)	19 (23.2)	0.177	16 (12.6)	24 (21.6)	0.082
ε2/ε4	1 (1.1)	0 (0)	0 (0)	/	1 (0.8)	0 (0)	/
ε3/ε3	54 (59.3)	37 (56.9)	52 (63.4)	0.715	78 (61.4)	65 (58.6)	0.653
ε3/ε4	24 (26.4)	16 (24.6)	9 (11)	0.029	31 (24.4)	18 (16.2)	0.148
ε4/ε4	0 (0)	0 (0)	1 (1.2)	/	0 (0)	1 (0.9)	/

SD, standard deviation; IQR, interquartile range; CA, carotid artery; TI, tortuosity index; TG, triglyceride; TC, total cholesterol; LDL-C, low-density lipoprotein cholesterol; ApoE, Apolipoprotein E.

### Multivariate logistic regression analysis

The variables demonstrated with statistical differences in univariate analysis, including age, gender, smoking history, alcohol consumption history, and the two target independent variables (ApoE genotype ε2/ε3 and ε3/ε4), were utilized into ordinal multiclassification logistic regression and binary logistic regression analyses to investigate the risk factors associated with ECA tortuosity.

After adjusting for age, female gender, and smoking, the ordinal multiclassification logistic regression for the tertile groups revealed that ε2/ε3 (OR = 1.116, 95% CI 0.550–2.264; *p* = 0.76) did not emerge as an independent risk factor, while ε3/ε4 (OR = 0.469, 95% CI 0.242–0.909; *p* = 0.025) demonstrated as a protective factor for ECA tortuosity. Neither ε2/ε3 (OR = 1.374, 95% CI 0.609–3.102; *p* = 0.444) nor ε3/ε4 (OR = 0.6, 95% CI 0.282–1.273; *p* = 0.183) showed the significant independent relationship to ECA tortuosity after adjusting for age, female gender, smoking, and alcohol consumption in binary logistic regression (Table 2).

**Table 2:** Multivariate logistic regression for ECA tortuosity.

Factors	The tertile groups of TI			The median groups of TI		
	OR	95% CI	*p* value	OR	95% CI	*p* value
Age	1.059	1.033–1.085	<0.001	1.059	1.029–1.090	<0.001
Female	4.345	2.286–8.258	<0.001	3.989	1.933–8.230	<0.001
Smoking	1.829	0.961–3.481	0.066	2.370	1.047–5.367	0.039
Alcoholism	/	/	/	0.570	0.227–1.432	0.231
ApoE genotype						
ε2/ε3	1.116	0.550–2.264	0.760	1.374	0.609–3.102	0.444
ε3/ε4	0.469	0.242–0.909	0.025	0.600	0.282–1.273	0.183

OR, odds ratio and CI, confidence interval.

### Correlation analysis between ApoE gene polymorphism and ECA tortuosity

**Figure 2:**
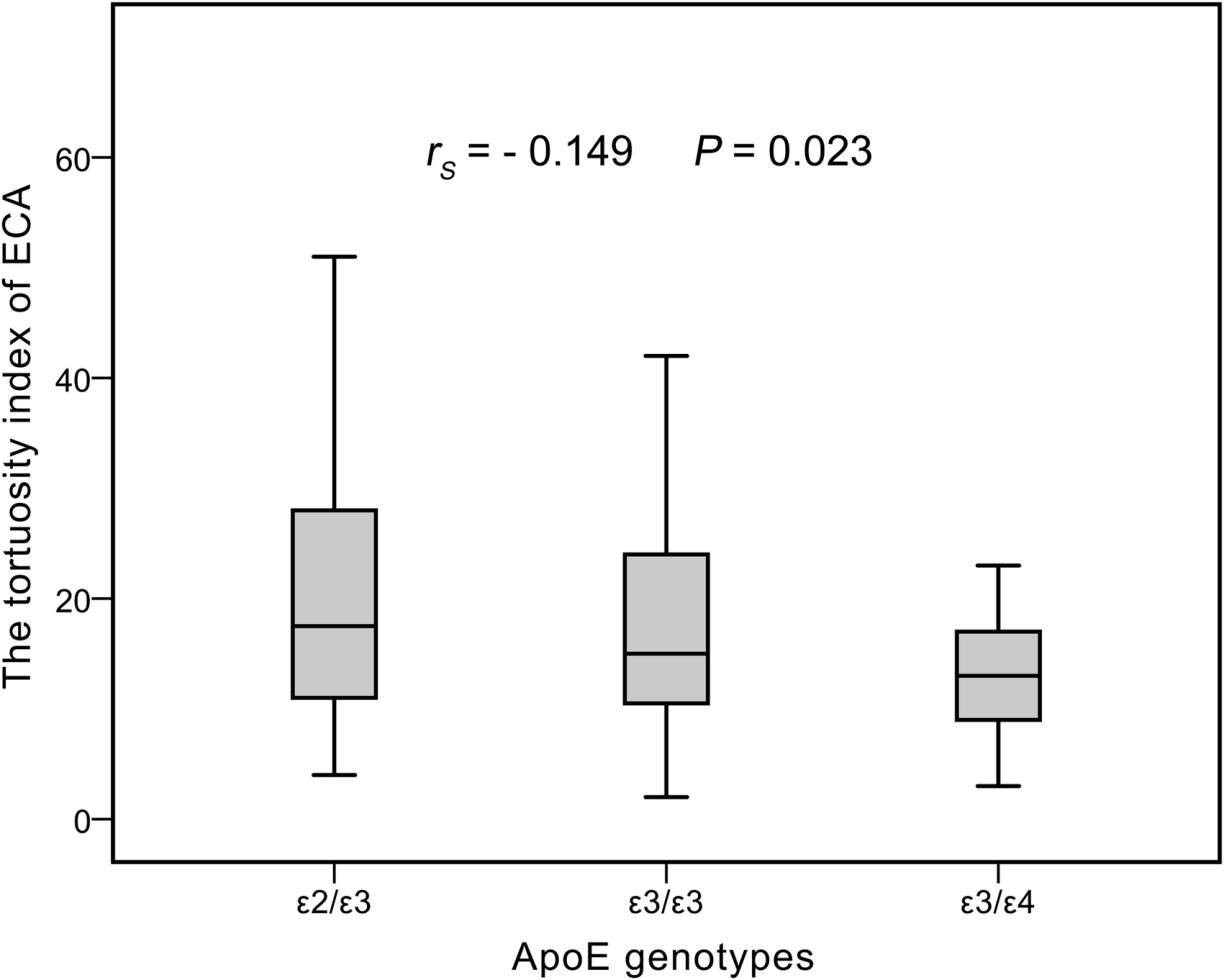
The distribution of ECA tortuosity index in ApoE genotypes and suggested a significant negative correlation. ECA, extracranial carotid artery; *r*_s_, spearman correlation coefficient.

The distribution of the ECA tortuosity index in ApoE genotypes (ε2/ε3, ε3/ε3, and ε3/ε4) is displayed in Figure 2. The Spearman’s rank correlation analysis revealed a significant negative correlation between the TI of ECA and ApoE genotypes (in sequential order: ε2/ε3, ε3/ε3, and ε3/ε4) (*r_S_* = −0.149, *p* = 0.023).

## Discussion

In this study, we have demonstrated a potential association between the ApoE gene polymorphism and the tortuosity of ECA in adult patients. Our findings indicated that individuals with the ApoE genotype ε3/ε4 exhibit an independent protective factor for ECA tortuosity. Furthermore, we have revealed a significant negative correlation between ECA tortuosity and three genotypes of ApoE (in sequential order: ε2/ε3, ε3/ε3, and ε3/ε4). Specifically, these results suggest that the ε4 allele may exert a protective effect on the tortuosity of ECA, while as for the ε3 allele, the most common wild-type of ApoE, has no significant influence on ECA tortuosity. Although the ε2 allele did not show the statistical significance in the multivariate analysis, according to the Spearman correlation analysis, the ε2 allele carriers still may exist a potential promotive mechanism for ECA tortuosity.

Apolipoprotein E, a basic protein rich in arginine, is one of the main apolipoproteins in plasma. ApoE gene can lead to a variety of diseases by affecting the metabolism of lipids in serum (16). The ε4 allele carriers have higher levels of cholesterol and low-density lipoprotein, while the ε2 allele carriers have lower levels of cholesterol and low-density lipoprotein. The ApoE2 isoform can reduce the level of low-density lipoprotein in plasma, so it is considered to play a protective role in atherosclerotic cardiovascular disease (ASCVD) (4). In addition, ApoE participates in regulating multiple information pathways in the central nervous system, including cholesterol/lipid homeostasis, synaptic function, glucose metabolism, neurogenesis, mitochondrial function, tau protein phosphorylation, neuronal atrophy, etc., thereby affecting cognitive function (17–19). The prevailing consensus suggests that ApoE4 serves as a critical genetic risk factor in the pathogenesis of neurodegenerative disorders, notably Alzheimer’s disease. On the contrary, ApoE2 is a genetic protective factor for Alzheimer’s disease (AD), and its exact mechanism is not yet clear (20). Furthermore, some previous studies related to neurodegenerative diseases suggest that ApoE2 may increases the risk of progressive supranuclear palsy (21, 22). In summary, the ApoE4 may plays an important role in the development of Aging process, while ApoE2 is a protective isoform for ASCVD and AD.

Although the pathogenesis is currently unclear, there are evidences to suggest the ECA tortuosity may association with ischemic stroke (23, 24), arterial dissection (25, 26), white matter hyperintensities (27, 28), connective tissue disease (29), and intracranial aneurysm (30). Considering the significant relationship between ECA tortuosity and age, it may be classified as a type of vascular morphological anomaly attributed to the process of aging. However, our research results surprisingly showed that ECA tortuosity seems to be positively correlated with the ε2 allele, a protective genetic gene for atherosclerosis and AD, and be negatively correlated with the ε4 allele, which is considered as a risk gene. Although whether arterial tortuosity is an independent risk factor for ischemic stroke or related to atherosclerosis remains controversial (31–33), our research results also found that common risk factors of stroke, such as dyslipidemia, hypertension, diabetes, smoking and alcohol consumption, could not be confirmed as the independent risk factors of ECA tortuosity. Apart from the factor of age, female participants exhibited a more pronounced tortuosity of the ECA, which may be attributed to their comparatively shorter average height when compared to males. Furthermore, although some genetic arterial diseases, such as Loeys-Dietz syndrome, Marfan syndrome, Aneurysm-osteoarthritis syndrome, can manifest as aortic or carotid artery tortuosity through the remodeling of vascular connective tissue (34), there are currently a lack of reports linking ApoE gene polymorphisms to these genetic connective tissue disorders. Overall, even though ECA tortuosity is considered to be associated with degenerative changes, there is still a significant discrepancy in the mechanism between ECA tortuosity and atherosclerosis. The underlying genetic factors and remodeling of vascular wall may both be involved in the pathogenesis of ECA tortuosity. However, it is still unclear how ApoE gene polymorphism affects ECA tortuosity, and further in-depth research is needed to explore the pathogenesis of vascular tortuosity.

There are several limitations in our study. First of all, there is a possibility of congenital bias due to this study is retrospective. Second, our research is a single center study and did not include the race factor of participants. It is uncertain whether there are differences in the results between different racial populations. Finally, the proportion of patients with ApoE genotype ε2/ε3 and ε3/ε4 were relatively small, which leads to certain difference in sample size between the groups, and it is necessary to increase the sample size, preferably through further research on big data.

## Conclusion

Our study suggested that the ApoE ε2 allele may be associated with increased tortuosity of ECA, whereas the ε4 allele may leads to be the protective factor. The ε3 allele, as the most prevalent wild-type in human, has not shown a significant influence on ECA tortuosity. The pathogenesis of ECA tortuosity may be associated with genetics and age-related vascular degenerative changes. In further research, we aim to verify the association between ApoE gene polymorphism and ECA tortuosity by expanding the sample size, conducting big data analysis, and including participants from diverse regions and ethnic backgrounds. Additionally, we anticipate more fundamental studies to elucidate the mechanisms underlying these research findings.

## Data Availability

The original contributions presented in the study are included in the article/supplementary material, further inquiries can be directed to the corresponding author.
